# Family Cluster Analysis of Severe Fever with Thrombocytopenia Syndrome Virus Infection in Korea

**DOI:** 10.4269/ajtmh.16-0527

**Published:** 2016-12-07

**Authors:** Jeong Rae Yoo, Sang Taek Heo, Dahee Park, Hyemin Kim, Aiko Fukuma, Shuetsu Fukushi, Masayuki Shimojima, Keun Hwa Lee

**Affiliations:** 1 Division of Infectious Diseases, Jeju National University School of Medicine, Jeju, South Korea; 2 Department of Microbiology and Immunology, Jeju National University School of Medicine, Jeju, South Korea; 3 Special Pathogens Laboratory, Department of Virology I, National Institute of Infectious Diseases, Japan

## Abstract

Severe fever with thrombocytopenia syndrome (SFTS) is tick-borne viral disease that was first suspected in China in 2009. The causative virus (SFTSV) was isolated in 2009 and reported in 2011, and SFTSV expanded its geographic distribution in 2012–2013, from China to South Korea and Japan. Most SFTSV infections occur through *Haemaphysalis longicornis*. However, SFTSV infection can also occur between family members, and nosocomial transmission of SFTSV is also possible through close contact with a patient. In this study, we first analyzed clinical, epidemiological, and laboratory data for SFTS patients and family members of an index patient in Korea. The S segment of SFTSV was amplified from the sera of three patients, and the S segment of SFTSV and IgG specific to SFTSV were detected in the serum from one family member; although this individual had no history of exposure to *H. longicornis*, she frequently had close contact with the index patient. In Korea, SFTSV infection among family members does not have to be reported, and we suggest that person-to-person transmission of SFTSV among family members is possible in Korea.

## Introduction

Severe fever with thrombocytopenia syndrome virus (SFTSV) is a tick-borne virus of the genus *Phlebovirus* and family *Bunyaviridae* that can cause hemorrhagic fever.^1^ SFTS was first confirmed in China in 2009 and was also reported and confirmed in South Korea and the western regions of Japan in 2013.^2^,^3^

In China, SFTS has an approximate case fatality rate (CFR) of 12%. Retrospective analysis of cases in Japan show an even higher average CFR, with four deaths of eight confirmed cases, and additional suspected cases still need to be confirmed.^4^ In South Korea, 122 patients were confirmed as having SFTSV infection, with 40 (a CFR of 32.8%) of these patients dying in Korea between July 2012 and August 2015.^5^

SFTSV has been detected in *Haemaphysalis longicornis*, which acts as a transmission host between animals and humans.^6^ Most SFTSV infections occur through *H. longicornis*.^6^ However, several studies have shown that SFTSV transmission can also occur between family members, and the nosocomial transmission of SFTSV is also possible through close contact with a patient.^7^–^9^

In this study, we describe a family cluster of SFTSV infections, including an analysis of household relationships and health-care workers (HCWs) in Korea.

## Materials and Methods

### Clinical investigation.

**Figure 1.**
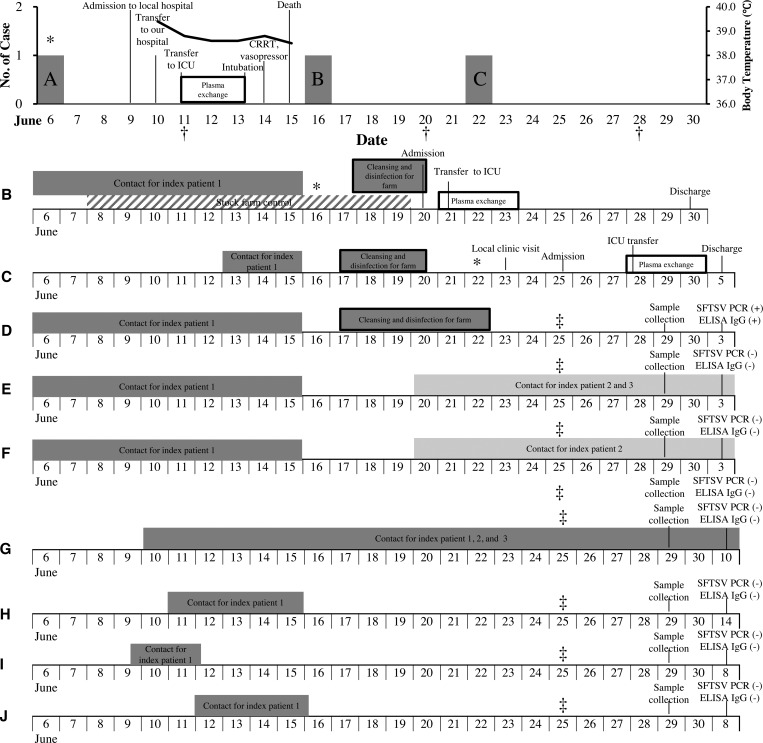
Epidemiologic curve of the family cluster, health-care workers and key events during the index patient's illness. A = Index patient (patient 1); B = patient 2 (son of A); C = patient 3 (son-in-law of A); D = wife of A; E = daughter of A; F = daughter-in-law of A; G = physician of patients 1, 2, and 3; H = physician of A; I = physician of A; J = physician of A. CRRT = continuous renal replacement therapy; ICU = intensive care unit. * Fever onset; † Severe fever with thrombocytopenia syndrome virus polymerase chain reaction confirmed; ‡ surveillance start for family and health-care workers.

The family cluster of SFTS first occurred in Jeju, Korea, in 2015. The index patient (patient 1) was admitted to Jeju National University Hospital on June 2015. Two additional patients, patient 2 (son of the index patient) and patient 3 (son-in-law of the index patient) were admitted to the same hospital on June 23, 2015, and June 25, 2015, respectively. We collected epidemiologic data, clinical features, and the clinical outcomes for each of the three patients (Figure 1

). Written informed consent to participate in the study was obtained from the patients, family members, and HCWs. The study was approved by the institutional review board (IRB) at Jeju National University Hospital (IRB file no. 2015-08-002).

### Epidemiologic investigation.

Jeju Island is the most prevalent region of SFTSV in South Korea (incidence: 1.3 patients/100,000 population).^5^

The family cluster of SFTS first occurred at the livestock farm on Jeju in 2015. The livestock farm is located at the northeast of Jeju, which is a high-prevalence region of SFTSV in Jeju and grazing land, woodlands, grass, trees, and forests. On June 25, 2015, we performed an epidemiological investigation and active surveillance of the family members and HCWs of the index patient. We interviewed the family members and HCWs who provided medical services to the patients. Serum samples obtained from the family members and HCWs were analyzed using reverse transcription polymerase chain reaction (RT-PCR) to test for SFTSV and enzyme-linked immunosorbent assay (ELISA) to test for IgG.

### RNA extraction.

Viral RNA was extracted from the sera of the SFTS patients, family members, and ticks from the index patient using a QIAamp Viral RNA Mini kit (Qiagen Inc., Mainz, Germany) according to the manufacturer's instructions. The extracted RNA was preserved in elution buffer at −70°C until introduction into a real-time RT-PCR assay.

### Real-time RT-PCR for molecular diagnosis.

Real-time RT-PCR of the partial S segment of SFTSV was performed for molecular diagnosis.^10^ The real-time RT-PCR mixture contained 8 μL of one-step RT-PCR premix, 7 μL of detection solution, and 5 μL of the RNA template in a total volume of 20 μL. PCR was performed under the following conditions: 30 minutes at 45°C, 10 minutes at 90°C, and 45 cycles of 15 seconds at 95°C and 30 seconds at 48°C.

### RT-PCR for sequencing and phylogenetic analysis.

RT-PCR of the S segment (1,480 base pairs [bp]) of SFTSV was performed for phylogenetic analysis.^11^ The RT-PCR mixture contained 8 μL of one-step RT-PCR premix, 7 μL of detection solution, and 5 μL of the RNA template in a total volume of 20 μL. PCR was performed under the following conditions: 30 minutes at 45°C, 10 minutes at 90°C, and 45 cycles of 15 seconds at 95°C and 30 seconds at 48°C.

### Sequencing and phylogenetic analysis of S segments of SFTSV.

For the partial S segments (1,480 bp), RT-PCR products were electrophoresed on a 1.2% agarose gel and purified using a QIAEX II gel extraction kit (Qiagen Inc.) according to the manufacturer's instructions. The RT-PCR products were then sequenced using a BigDye Terminator Cycle Sequencing kit (PerkinElmer Applied Biosystems, Warrington, United Kingdom).^11^ The sequences of the S segments were aligned using the multiple-alignment algorithm in the MegAlign program (Windows version 3.12e; DNASTAR, Madison, WI) and the ClustalX program. Based on the aligned sequences, phylogenetic analyses were conducted in MEGA6, and phylogenetic trees were constructed using the maximum likelihood method. The bootstrap consensus tree inferred from 1,000 replicates was used to represent the evolutionary history of the taxa analyzed.^11^,^12^

### Antibody detection in sera.

An ELISA was performed as previously described, except for antigen preparation.^13^–^16^ Antigen preparation for the IgG and IgM ELISA was performed as follows. Huh7 cells were infected with SFTSV strain HB29 (multiplicity of infection = 0.1) and incubated at 37°C for 48 hours. The cells were collected and washed with phosphate-buffered saline (PBS), before being lysed with PBS solution containing 1% NP-40. The cell lysates were centrifuged at 8,000 rpm for 10 minutes at 4°C, and the supernatant was collected as a source of SFTSV antigen for the IgG ELISA. Huh7 cell lysates prepared without infection were used as a negative control. A Nunc-Immuno Plate (Thermo Fisher Scientific Inc., Waltham, MA) was coated with the predetermined optimal quantity of cell lysates from SFTSV-infected or uninfected Huh7 cells (diluted 1:800 with PBS) and incubated at 4°C overnight. The following procedure was performed in the same way as described previously.^13^ The cutoff value was set as the average value of the control sera (sera from healthy donors) plus three times the standard deviation (SD = mean + 3 × SD). A sample was considered positive if it yielded an OD_405_ value above the cutoff value.

### Neutralization test.

50% focus reduction neutralization titer (FRNT50) was determined as follows: approximately 100 focus-forming units of SFTSV were mixed with serially diluted sera and incubated for 1 hour at 37°C, then inoculated onto confluent monolayers of Vero cells in 12 multiple-well plates for additional 1 hour at 37°C. The inoculums were removed and the cells washed once with Dulbecco's modified eagle's medium (DMEM) containing 2% fetal calf serum (FCS) and cultured at 37°C in DMEM containing 2% FCS and 1% methylcellulose. Seven days later, inoculated cells were fixed with 10% formalin and exposed to ultraviolet (UV) light to inactivate SFTSV. The cells were treated with 0.1% Triton X-100 (Sigma-Aldrich, St. Louis, MO) followed by staining with rabbit anti-N antibody (Fukuma and others) and horse radish peroxidase–labeled secondary antibody. Focus of inoculated cells were visualized by using peroxidase stain DAB Kit (Brown Stain; Nacalai Tesque, Kyoto, Japan) and counted. FRNT50 were determined as reciprocal of the highest dilution which showed less than 50% of focus numbers obtained without serum.^17^

### Immunofluorescence assay in sera.

Vero cells were obtained from the American Type Culture Collection and inoculated with SFTSV strain HB29. Three days later, the inoculated cells were detached by trypsin/ethylenediaminetetraacetic acid treatment and mixed with mock-inoculated Vero cells at a ratio of 1:3. After washing with PBS, the cells were smeared onto multiple-well slides (Matsunami Glass, Osaka, Japan), fixed with acetone, exposed to UV light, and used as the immunofluorescence assay (IFA) antigen. Sera were serially diluted two times with PBS starting with a 1:20 dilution and spotted onto the slides. After 1-hour incubation at 37°C, the slides were washed with PBS. The slides were further incubated with Alexa Fluor^®^ 488-conjugated goat anti-human IgG (H+L) (1:200 dilution, Life Technologies) or fluorescein isothiocyanate–goat anti-human IgM (μ chain) (1:200 dilution, Invitrogen) for 1 hour at 37°C. The slides were washed with PBS and observed under a fluorescence microscope (Olympus, Tokyo, Japan).^4^

## Results

### Case descriptions.

Figure 1 shows the case progression for the index patient (patient 1, A), the cluster of other family members with confirmed SFTSV, and a contact history of additional family members. The index patient (A), a healthy 74-year-old male, had been unwell with fever, myalgia, and headache. His occupation was a cattle rancher. A physical examination recorded the following vital statistics: 39.4°C body temperature, 149/90 mm of Hg blood pressure, 90/minutes pulse rate, and 22/minutes respiratory rate. The patient discovered a crusty round erythematous cutaneous lesion suspected to be a tick bite on his right axilla and presented with a skin rash covering his whole body. In addition, ticks were found on his clothing. Initial complete blood count revealed neutropenia (2,100/mm^3^) and thrombocytopenia (47,000/mm^3^). Aspartate aminotransferase and alanine aminotransferase were mildly elevated at 157 and 56 IU/L, respectively. In addition, creatinine phosphokinase and lactate dehydrogenase levels were elevated. On day 2 at the hospital, the presence of SFTSV was confirmed by RT-PCR. As dictated by hospital SFTS protocol, the patient received a plasma exchange due to deterioration in his laboratory results.^18^ However, on hospital day 3, his mental status altered to a stupor, and he progressed to multiorgan failure and hypotension the following day. Mechanical ventilation and continuous renal replacement therapy were initiated on hospital day 6, despite intensive medical treatment and the absence of hemorrhagic tendency. Patient 2 (B), a 45-year-old office worker and the eldest son of the index patient, presented to the emergency department on June 20, 2015, with a 4-day history of fever and myalgia. He had worked his father's job during the hospitalization of the index patient. He also had close contact with his father before his father's hospitalization, but did not come in contact with blood or bloody body fluid. On June 16, 2015, patient 2 developed a fever after his father's funeral. He had been working on cleaning and disinfecting his father's farm equipment since June 18, 2015, in an attempt to prevent additional transmission. He suspected SFTS during his visit to the ranch, as he observed a tick lesion on his right thigh. A molecular diagnosis for SFTSV was confirmed on hospital day 1 (for patient 2). Plasma exchange was started immediately after the SFTS diagnosis. His general condition and laboratory findings improved, and he was discharged on hospital day 10 (for patient 2). Patient 3 (C), a 45-year-old car engineer and son-in-law of the index patient, presented with fever, myalgia, and rigor on June 22, 2015. He was previously healthy and had worked on his father-in-law's ranch at the same time as patient 2. He was also admitted with suspicion of SFTS on June 25, 2015, as there was a tick lesion on his right axilla. As expected, RT-PCR confirmed SFTSV, and plasma exchange was performed as per protocol. He was discharged from the hospital on day 11 (for patient 3).

### Family cases of SFTS and epidemiologic investigation.

We began surveillance of the family members of the index patient on June 25, 2015. Three additional family members were identified as having had contact with the SFTS patients (Figure 1). The wife (D) of patient 1 lived in the same household as the index patient (A), his daughter-in-law (F) lived in the same household as patient 2 (B), and his daughter (E) lived in the same household as patient 3 (C). The wife of patient 1 worked with patient 1 on the livestock farm for many years. She did not exhibit any infection-related symptoms and did not have evidence of tick bite lesions. The daughter and daughter-in-law had never been exposed to the livestock on the farm. Neither presented any symptoms (Figure 1).

An additional four HCWs (three physicians and one nurse) also came into contact with the infected patients, but all used personal protective equipment (mask and gloves). We interviewed all additional contacts and collected blood samples. All the HCWs who had contact with the patients remained healthy (Figure 1).

The S segment of SFTSV was confirmed in three patients (A, B, and C) and family 1 (D) by real-time RT-PCR. The patients may have been infected with the same SFTSV (the similarity was 100% [124/124]) (Supplemental Figure 1).^10^

**Figure 2.**
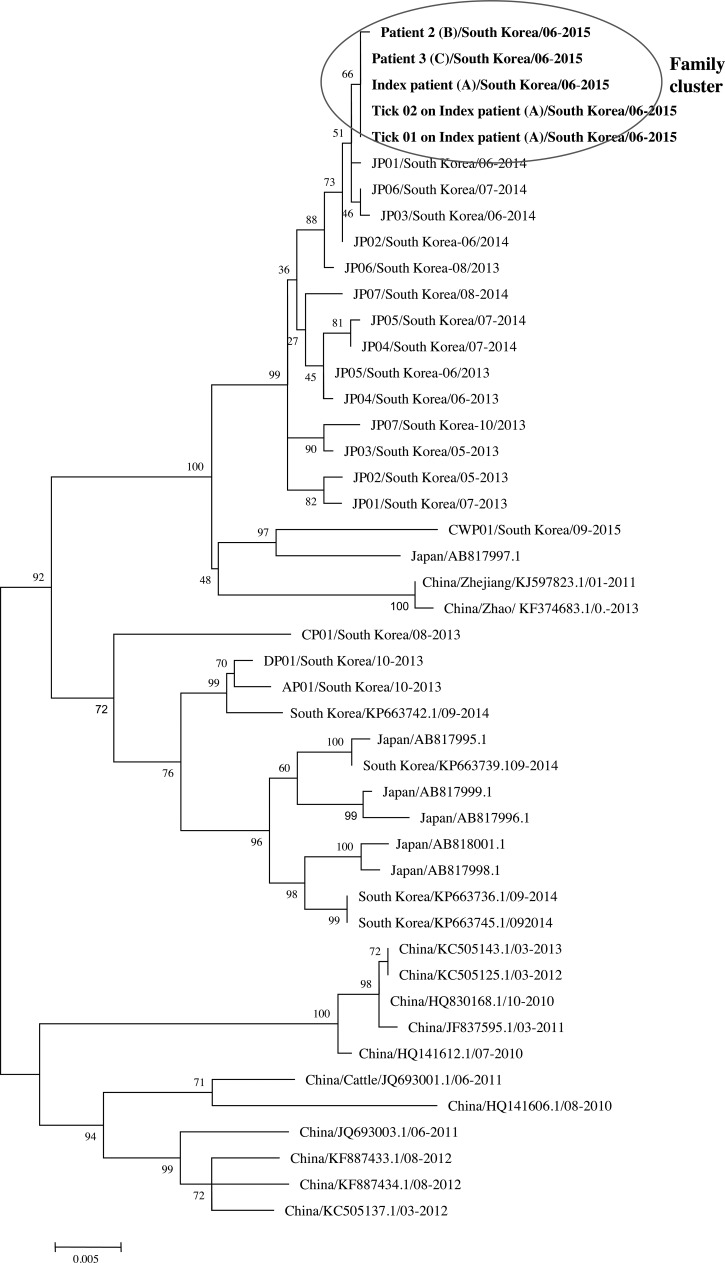
Phylogenetic tree constructed based on the S segment. The tree was constructed using the maximum likelihood method with MEGA 6.^12^ The S sequences of the family cluster and ticks in this study are shown in bold. Index patient/South Korea/06-2015 was amplified from the index patient (A), patient 2/South Korea/06-2015 was amplified from patient 2 (B), and patient 3/South Korea/06-2015 was amplified from patient 3 (C) in June 2015. Tick 01 on index patient/South Korea/06-2015 was amplified from a tick on the index patient, and Tick 02 on index patient/South Korea/06-2015 was also amplified a from tick on the index patient. The S sequence data for the viruses identified in China, South Korea, and Japan were obtained from NCBI/BLAST.

A phylogenetic tree showed that the index patient 1 (A), patient 2 (B), and patient 3 (C) were confirmed to have the same SFTSV S segment and may have been infected with the same SFTSV (the similarity was 100%). In addition, the S segment of the ticks found on the index patient exhibited a similarity of 99% (Figure 2

).

Serum samples collected from the three patients (A, B, and C), three family members (D, E, and F), and four HCWs were tested for IgG and IgM to SFTSV using ELISA and IFA.

**Figure 3.**
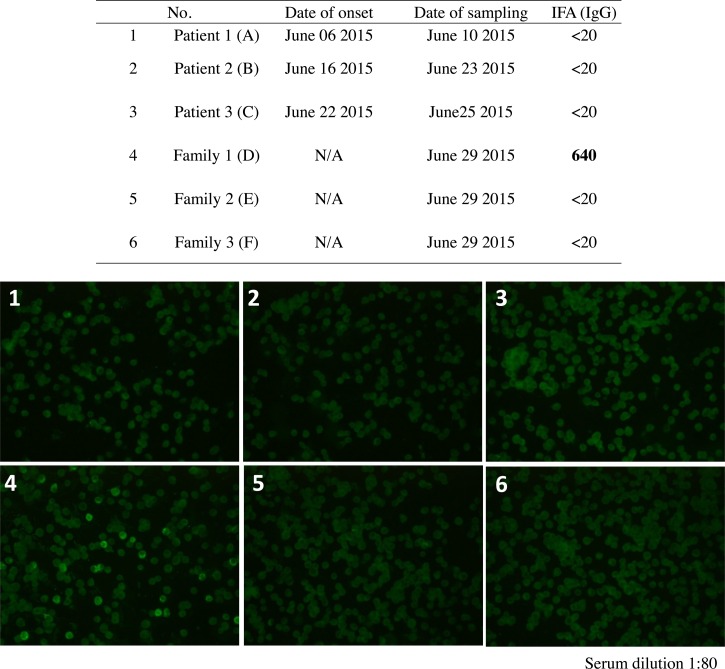
Immunofluorescence assay for IgG in a family cluster in 2015. Serum dilution 1:80.

The results of the ELISA and IFA used to detect IgG showed no SFTSV-specific IgG in the sera of the three patients, two family members, or four HCWs, while the results of the ELISA used to detect IgM showed no SFTSV-specific IgM in the sera of the patients, their family members, or any of the HCWs (Tables 1 and 2, and Figure 3

).

However, SFTSV-specific IgG was detected on June 29, 2015, in the serum from one family member (family 1, D), who is the wife of the index patient and had close contact with the fatally ill patient (index patient, A) 23 days after the onset of illness in the index patient. She exhibited increasing levels of SFTSV-specific IgG on August 19, 2015, 73 days after onset in the index case. She had no history of tick bites and never presented symptoms of SFTS (Tables 1 and 2, Figure 3).

## Discussion

SFTS is a viral disease that infects humans primarily through tick bites.^1^ Transmission from person to person has been confirmed in both family members and HCWs, and asymptomatic infection via person-to-person contact has also been reported.^7^–^9^

In this study, we describe a family cluster of three confirmed cases and one probable case of SFTSV infection, probably transmitted through close contact with a patient, using epidemiology, serology, and phylogenetic analysis.

The S segment of SFTSV was confirmed in the sera of three patients and one family member. SFTSV-specific IgG was not detected in the sera of three patients or two family members. However, SFTSV-specific IgG was detected in the serum of one family member (the wife of the index patient), who had close contact with the fatally ill patient (index patient) (Tables 1 and 2, Figure 3).

Epidemiological data indicated that the three symptomatic cases, including the index patient, had a history of exposure to ticks at the same location, whereas the asymptomatic family member had no history of exposure to *H. longicornis* at this location (Figure 1).

In this study, we suggest that the index patient (the fatally ill patient) was infected by a virus-bearing tick and that a family member may have been infected via close contact with the index patient.

In conclusion, the transmission of SFTSV from person to person could have occurred among family members. Therefore, it is important to control the additional SFTSV infection of patient relatives and other affected populations, and epidemic research and prevention management should consider these possible channels of infection.

## Supplementary Material

Supplemental Figures.

## Figures and Tables

**Table 1:** The clinical and laboratory characteristics of family cluster of SFTSV infection in 2015, Korea

Patients	Index patient Patient 1 (A)	Patient 2 (B)	Patient 3 (C)	Family 1 (D)	Family 2 (E)	Family 3 (F)	Two ticks on index patient (A)
Relationship with index patient	NA	Son	Son-in-law	Wife	Daughter	Daughter-in-law	NA
Occupation	Rancher	Office job	Technician	Rancher	Housewife	Housewife	NA
Contact history of livestock and forest land	Yes	Yes	Yes	Yes	No	No	NA
Close contact with index case before admission	NA	No	No	Frequent	No	No	NA
Close contact with index case after admission	NA	Yes	Yes	Yes	No	No	NA
Date of onset	June 06, 2015	June 16, 2015	June 22, 2015	NA	NA	NA	NA
Date of admission	June 10, 2015	June 23, 2015	June 25, 2015	NA	NA	NA	NA
Date of sampling	June 10, 2015	June 23, 2015	June25, 2015	June 29, 2015	June 29, 2015	June 29, 2015	NA
Clinical diagnosis of SFTSV infection	Yes	Yes	Yes	No	No	No	NA
Temperature (°C)	39.4	37.9	39.4	NA	NA	NA	NA
ANC	1,554	2,310	1,062	NA	NA	NA	NA
Platelet (×10^3^/μL)	47	118	93	NA	NA	NA	NA
AST (IU/L)	157	72	22	NA	NA	NA	NA
ALT (IU/L)	66	97	16	NA	NA	NA	NA
A history of exposures to tick	Yes	Yes	Yes	NA	NA	NA	NA
ICU admission	Yes	Yes	Yes	NA	NA	NA	NA
Disseminated intravascular coagulation	Yes	No	No	NA	NA	NA	NA
Mechanical ventilation	Yes	No	No	NA	NA	NA	NA
Vasopressor	Yes	No	No	NA	NA	NA	NA
Plasmapheresis	Yes	Yes	Yes	NA	NA	NA	NA
Amplification of S segment of SFTSV by real-time PCR	+	+	+	+	–	–	+
Identity of partial sequences of S segment among case A, B, C, D, and ticks from index case (A)	100%	100%	100%	100%	NA	NA	99%
IgG capture ELISA	–	–	–	+	–	NA	NA
Admission day	5	10	11	NA	NA	NA	NA
Clinical outcome	Death	Recovered	Recovered	No symptom	No symptom	No symptom	NA

ALP = alkaline phosphatase; ALT = alanine aminotransferase; ANC = absolute neutrophil count; AST = aspartate aminotransferase; ELISA = enzyme-linked immunosorbent assay; NA = not available; RT-PCR = reverse transcription polymerase chain reaction; SFTSV = severe fever with thrombocytopenia syndrome virus.

**Table 2:** Detection of anti-SFTSV antibody in a family cluster in 2015, Korea

No.	Family cluster	Neutralization test	IgG						
			IgG ELISA OD value (SFTSV infected-Mock) > cutoff value					IgM	
			100	400	1,600	6,400	IFA (IgG)	IgM capture ELISA	IFA (IgM)
1	Patient 1 (A)	< 20	−	−	−	−	< 20	−	< 20
2	Patient 2 (B)	< 20	−	−	−	−	< 20	−	< 20
3	Patient 3 (C)	< 20	−	−	−	−	< 20	−	< 20
4	Family 1 (D)	> 320	+	+	+	+	640	−	< 20
5	Family 2 (E)	< 20	−	−	−	−	< 20	−	< 20
6	Family 3 (F)	< 20	−	−	−	−	< 20	−	< 20

ELISA = enzyme-linked immunosorbent assay; IFA = immunofluorescence assay; SFTSV = severe fever with thrombocytopenia syndrome virus.
