# Supplementary material for: Family Cluster Analysis of Severe Fever with Thrombocytopenia Syndrome Virus Infection in Korea
Source: Am J Trop Med Hyg. 2016 Dec 7;95(6):1351–7. doi: 10.4269/ajtmh.16-0527 (PMC5154449; doi:10.4269/ajtmh.16-0527)
Supplement: Supplementary file 1 [file SD3.pdf]

A

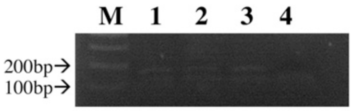

B

ACCTCTTAGACCCTGAGTTTGACATTTTCCCTGATGCCTTGACGATCTTATTGCCTCGGGTCA

GGCAAAGACAATGATGAACTTTGTATCTCTCACCCAATCATCTCCACCTGTCTCCTTCAGA

Supplemental Figure 1. (**A**) Amplification of the partial S sequences of severe fever with thrombocytopenia syndrome virus from family 1 (D) by real-time reverse transcription polymerase chain reaction[10] and (**B**) partial S sequences. Lane M = marker DNA (25- and 100-base-pair mixed DNA ladder); 1 = family 1; 2 = family 1; 3 = family 1; 4 = negative control.

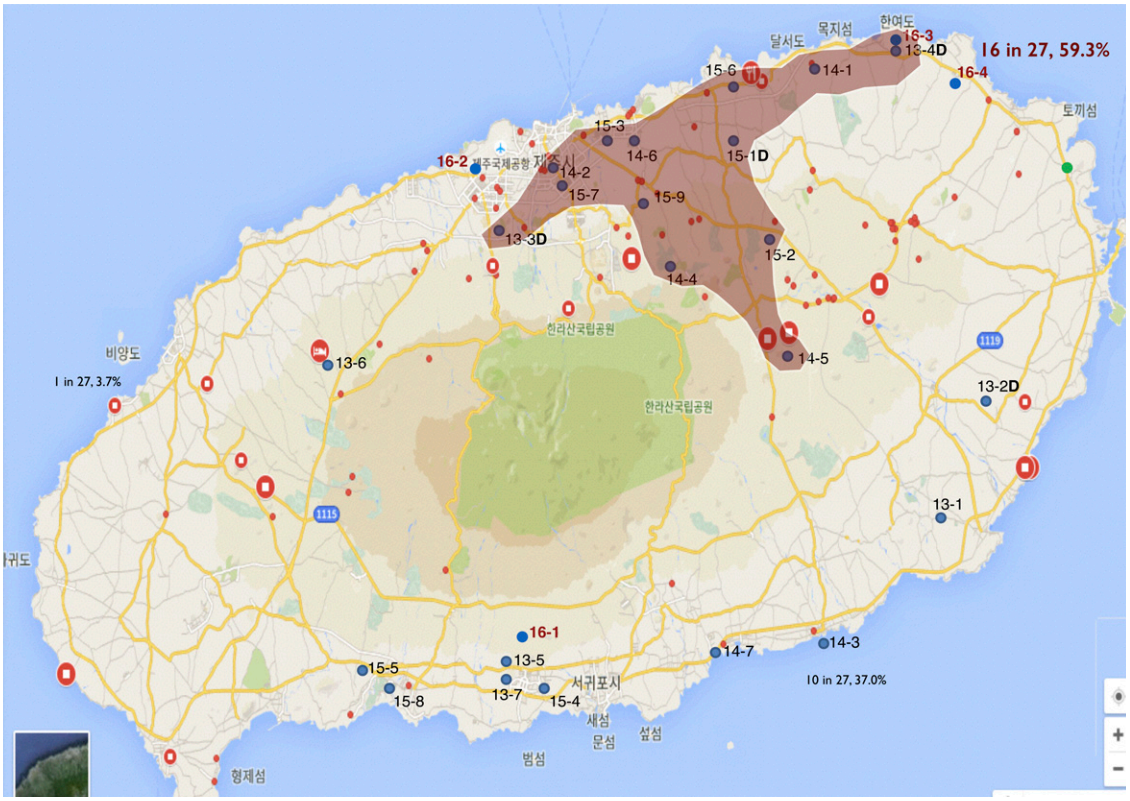

Supplemental Figure 2. Geographical distribution of severe fever with thrombocytopenia syndrome cases in Jeju, Korea, from May 2013 to August 2016 (n = 27).
